# Data on the effect of CdS on the lateral collection length of charge carriers for Cu(In,Ga)Se_2_ solar cells with mesh transparent conducting electrodes

**DOI:** 10.1016/j.dib.2020.105352

**Published:** 2020-02-28

**Authors:** Sangyeob Lee, Jiseong Jang, Joon Sik Park, Yong-Jun Oh, Choong-Heui Chung

**Affiliations:** Department of Materials Science and Engineering, Hanbat National University, Daejeon, 34158, Republic of Korea

**Keywords:** Lateral collection length, Lateral photocurrent, CdS, Mesh TCE, CIGS, Solar cells

## Abstract

Mesh transparent conducting electrodes (TCEs) have been successfully employed to Cu(In,Ga)Se_2_ (CIGS) solar cells (Lee et al., 2018; Jang et al., 2017; Lee et al., 2020) [1-3]. Lateral motion of charge carriers is necessarily required for the carriers to be collected in CIGS solar cell cells having mesh TCEs. Lateral collection length of carriers can be obtain based on the lateral photocurrent values measured in custom designed CIGS test structures, which in turn enables to determine an optimum design of mesh TCEs for these CIGS solar cells (Lee et al., 2019) [4]. In a standard CIGS solar cell, a CdS layer is required to be fully cover the CIGS whole surface. However, it is not the case for mesh TCE based CIGS solar cells (Chung, 2019) [5]. The presence or absence of the CdS layer on the CIGS/Mo planar stack alters the traveling path of the charge carriers, which in turn will affect the lateral photocurrent values. Therefore, it will be helpful to know the effect of the presence or absence of the CdS layer on the lateral photocurrents in mesh TCE based CIGS solar cells.

**Table:** Specifications Table

Subject	Electrical engineering
Specific subject area	Solar cells
Type of data	Table and Figures
How data were acquired	Keithley 2401 source meter under light illumination, a UV–vis spectrometer, and an optical power meter.
Data format	Raw, and analyzed
Parameters for data collection	Wavelength of incident light, and the presence or absence of a CdS layer on a CIGS/Mo planar stack.
Description of data collection	Lateral photocurrent values were measured for short-circuit conditioned two CIGS devices as function of wavelength of incident light. One is a planar stack of CdS/CIGS/Mo, and the other one is a planar stack of CIGS/Mo. Disk-shaped opaque top contacts were formed onto the both stacks.
Data source location	Hanbat National University, Daejeon 34,158, Republic of Korea
Data accessibility	Raw data related to Fig. 3, Fig. 4 are available within this article as a supplementary file.
Related research article	Author's name: Sangyeob Lee et al. Title: Determination of the lateral collection length of charge carriers for silver-nanowire-electrode-based Cu(In,Ga)Se_2_ thin-film solar cells Journal: Solar Energy https://doi.org/10.1016/j.solener.2019.01.059

**Table:** 

**Value of the Data** • Lateral collection length of the carriers, which can be determined from the lateral photocurrent values, is necessary to properly design mesh TCEs for CIGS solar cells. • CdS can be formed either on the CIGS whole surface or on the CIGS surface just below a mesh TCE for mesh TCE based CIGS solar cells. Lateral collection length is dependent on how CdS cover the CIGS surface because the presence or absence of CdS onto CIGS/Mo alters traveling paths of photogenerated charge carriers. The effect of CdS on the lateral collection length will help researcher to design mesh TCE and mesh TCEs based CIGS device structures. • It will be helpful to know the effect of the presence or absence of CdS on the wavelength-dependent lateral photocurrents to have a better understanding on the traveling paths of charge carriers in various solar cell structures.

## 1 Data

**Fig. 1:**
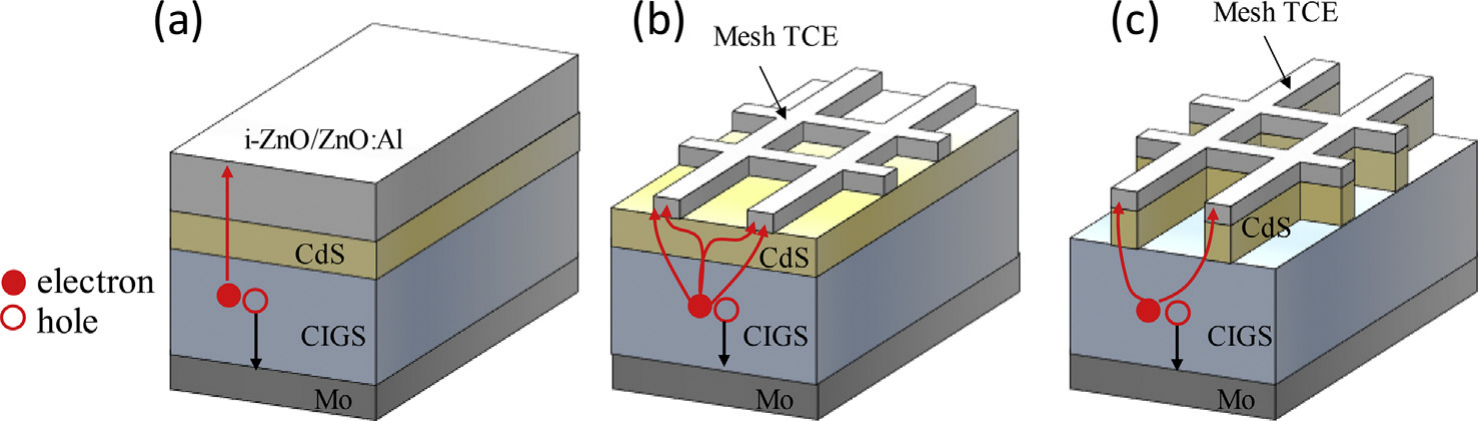
Schematics of (a) a standard CIGS solar cell, (b) a mesh TCE based CIGS solar cell with the CdS covering the CIGS whole surface, and (c) a mesh TCE based CIGS solar cell with the CdS layer covering the CIGS just below a mesh TCE.

**Fig. 2:**
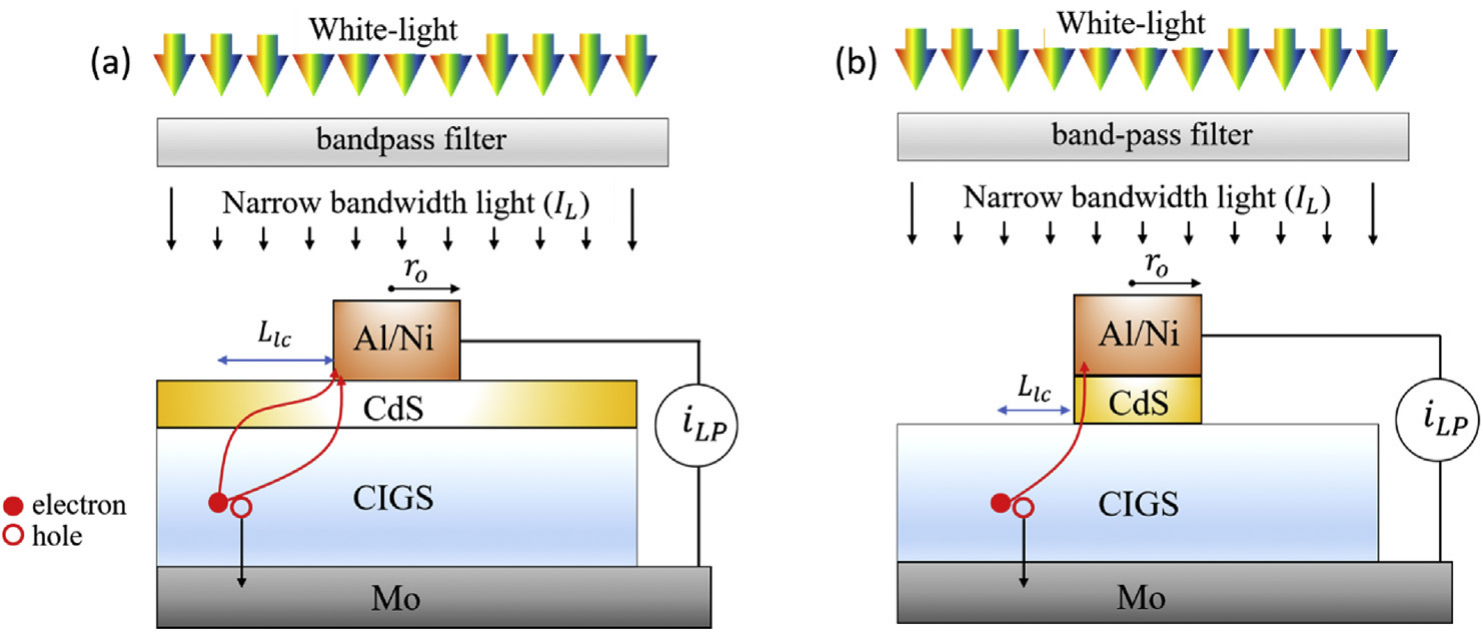
Schematics of the measurement of lateral photocurrent for two CIGS test structures. (a) A CdS/CIGS/Mo planar stack with a disk-shaped Al/Ni top contact, (b) a CIGS/Mo planar stack with a disk-shaped Al/Ni/CdS top contact.

**Fig. 3:**
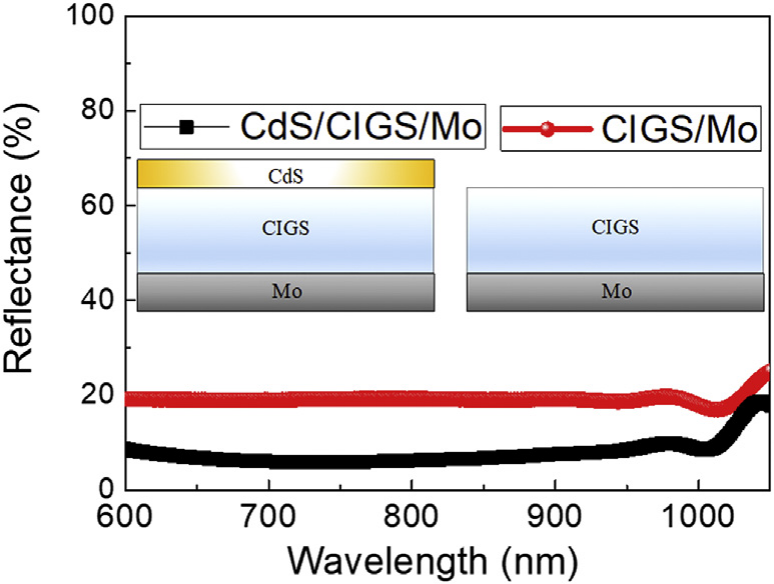
The reflectance of the light off CdS/CIGS/Mo and CIGS/Mo.

**Fig. 4:**
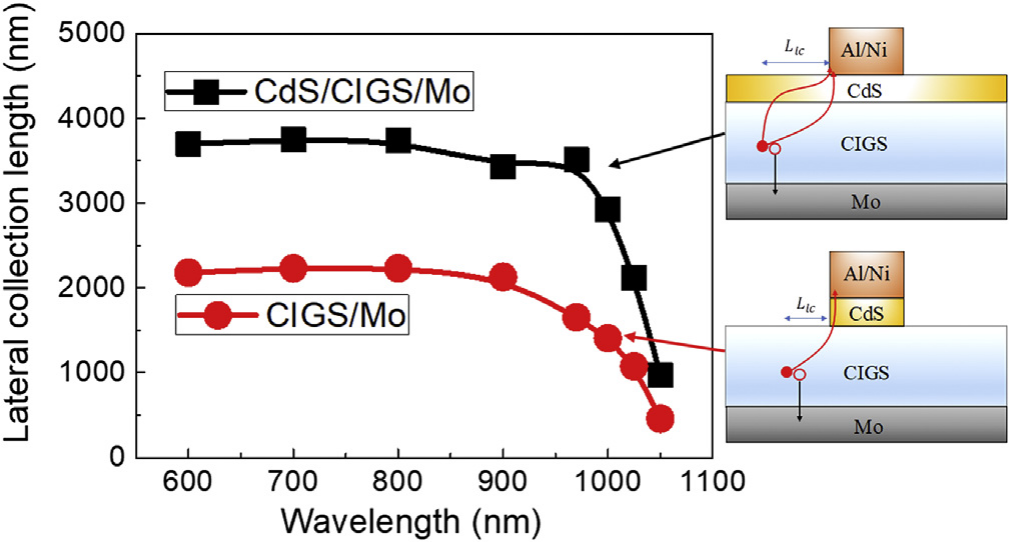
The lateral collection lengths of charge carriers as a function of wavelength of incident light for CdS/CIGS/Mo and CIGS/Mo planar stacks.

Photogenerated charge carriers travel in vertical directions to be collected in a standard CIGS solar cell (Fig. 1a). Mesh TCE based CIGS solar cells have shown comparable to or even better performance than standard CIGS solar cells [[1], [2], [3]]. Mesh TCE based CIGS solar cells necessarily need lateral motion of charge carriers in the empty space of the mesh TCE [4]. In addition, although a CdS layer covers the CIGS whole surface in a standard CIGS solar cell, it is not the case for mesh TCE based CIGS solar cells. As shown in Fig. 1b and c, the CdS layer can be formed either on the CIGS whole surface or on the only overlapped region of the CIGS and a mesh TCE [5]. The presence or absence of the CdS layer in the empty space of the mesh TCE alters the traveling paths of photogenerated electrons as shown in Fig. 1b and c [4,6]. The knowledge on the effect of the CdS layer on the lateral collection length of the carriers, which can be determined from the lateral photocurrent values, will be helpful to properly design mesh TCEs depending on CIGS device structures (Fig. 1b and c). Therefore, the lateral photocurrents were measured for the two CIGS test structures as shown in Fig. 2a and b. One is a CdS/CIGS/Mo planar stack with a disk-shaped Al/Ni top contact. The other one is a CIGS/Mo planar stack with a disk-shaped Al/Ni/CdS top contact. In order to measure lateral photocurrents at various wavelengths of incident light, optical bandpass filters are placed between the samples and a white-light source (Fig. 2). Data related to Fig. 3, Fig. 4 are available within this article as a supplementary file.

Lateral collection length ($L_{lc}$) can be determined by Eq. (1) [4]:

(1) $$L_{lc}=\frac{\left(\frac{hc}{\lambda }\right)\left(\frac{i_{lp}}{I_{L}2\pi r}\right)}{e\left(1-R_{f}\right)}$$

where *h* is the Planck constant, *c* is the speed of light, and λ is the wavelength of light, $I_{L}$ is the intensity of light, and r is the radius of the disk-shaped top contact, being 1 mm, and *e* is the electron charge. $R_{f}$ is the reflectance of the light off CdS/CIGS/Mo or CIGS/Mo, and shown in Fig. 3. Eight optical bandpass filters were used, and their center wavelength (CWL) and full width half maximum (FWHM) are summarized in Table 1. The measured values of $R_{f}$, $I_{L}$ and $i_{LP}$ are also summarized in Table 1.

**Table 1:** Center wavelength (CWL) and full width half maximum (FWHM) of the employed optical bandpass filters, the intensities ($I_{L}$) of light, reflectance ($R_{f}$) and lateral photocurrent ($i_{LP}$) values for two CIGS test structures. One is a CdS/CIGS/Mo planar stack with a disk-shaped Al/Ni top contact, and the other one is a CIGS/Mo planar stack with a disk-shaped Al/Ni/CdS top contact.

Bandpass filter		${\text{I}}_{\text{L}}$(W/m^2^)	CdS/CIGS/Mo		CIGS/Mo	
CWL (nm)	FWHM (nm)		${\text{R}}_{\text{f}}$(%)	${\text{i}}_{\text{LP}}$(nA)	${\text{R}}_{\text{f}}$(%)	${\text{i}}_{\text{LP}}$(nA)
600	50	145.4	8.6	1494	19.2	778
700	50	126.0	6.0	1573	19.0	807
800	50	99.2	6.2	1410	19.2	725
900	50	163.0	7.6	2354	19.1	1280
970	10	6.7	9.6	105	19.5	44
1000	10	15.1	8.7	204	17.9	88
1025	50	44.8	13.2	429	18.3	204
1050	10	6.9	17.9	29	25.0	13

Fig. 4 shows the lateral collection length $\left(L_{lc}\right)$ values as a function of wavelength of incident light for CdS/CIGS/Mo and CIGS/Mo planar stacks. The $L_{lc}$ values for the CdS/CIGS/Mo planar stack were approximately 3430–3750 nm in the range of 600–970 nm of wavelength, and the decreased to 972 nm with further increase of wavelength to 1050 nm. The $L_{lc}$ values for the CIGS/Mo planar stack were approximately 2130–2230 nm in the range of 600–900 nm of wavelength, and the decreased to 461 nm with further increase of wavelength to 1050 nm. In overall, the $L_{lc}$ values for the CdS/CIGS/Mo planar stack are approximately 1.6–2.1 times higher than those for the CIGS/Mo planar stack.

## 2 Experimental design, materials, and methods

### 2.1 Fabrication of CIGS test structures

The first CIGS test pattern was designed for photogenerated carriers to travel through both the CIGS and the CdS thin films (Fig. 2a). It has a disk-shaped Al/Ni with a radius (*r*_*o*_) of 1 mm on planar stacked CdS/CIGS/Mo. The second CIGS test pattern was designed for photogenerated carriers to be collected only through the CIGS film (Fig. 2b). It is a CIGS/Mo planar stack with a disk-shaped Al/Ni/CdS top contact. The second CIGS test pattern was achieved by removing the CdS of the first CIGS pattern using wet chemical etching. Mo (1 μm) was prepared by DC-sputtering technique on a soda-lime glass, CIGS (2 μm) was prepared by three stage co-evaporation method, chemical bath deposition was employed to prepare CdS (60 nm), and Al (1 μm)/Ni (50 nm) was e-beam evaporated through a shadow mask with a disk-shaped hole. More details on the preparation methods can be found elsewhere [7].

### 2.2 Characterization of CIGS test structures

The reflectance of light off CdS/CIGS/Mo and CIGS/Mo planar stacks were measured using a UV–Visible spectrometer (UV-2600, Shimadzu) equipped with ISR-2600 Plus integrating sphere. Now, we describe the measurement of the lateral photocurrent values as a function of wavelength of light for the CIGS test patterns. The white-light was generated by a solar simulator (Model 11002 SunLite, Abet technologies). Narrow bandwidth light was generated by passing the white-light through optical bandpass filters. Wavelength of incident light was adjusted by placing an appropriate optical bandpass filter between the samples and the white-light source. The light intensities were measured using an optical power meter (S120VC, PM100USB, Thorlabs). The lateral photocurrents were measured at a short-circuit condition between Al and Mo using Keithley 2401 source meter.
