# Supplementary material for: Data on the effect of CdS on the lateral collection length of charge carriers for Cu(In,Ga)Se2 solar cells with mesh transparent conducting electrodes
Source: Data Brief. 2020 Feb 28;29:105352. doi: 10.1016/j.dib.2020.105352 (PMC7063106; doi:10.1016/j.dib.2020.105352)
Supplement: Multimedia component 1 [file mmc1.pdf]

Fig. 3

wavelength (nm)	Reflectance (%)	
	CdS/CIGS/Mo	CIGS/Mo
600	8.588	19.199
601	8.542	19.194
602	8.496	19.193
603	8.45	19.183
604	8.406	19.173
605	8.353	19.163
606	8.31	19.164
607	8.26	19.16
608	8.216	19.158
609	8.177	19.157
610	8.134	19.157
611	8.096	19.146
612	8.052	19.14
613	8.012	19.128
614	7.969	19.123
615	7.928	19.112
616	7.887	19.105
617	7.85	19.094
618	7.818	19.091
619	7.785	19.079
620	7.744	19.075
621	7.712	19.075
622	7.671	19.075
623	7.629	19.073
624	7.596	19.08
625	7.555	19.076
626	7.526	19.078
627	7.491	19.083
628	7.458	19.073
629	7.427	19.07
630	7.395	19.066
631	7.367	19.061
632	7.335	19.05
633	7.304	19.06
634	7.27	19.051
635	7.228	19.058
636	7.201	19.055
637	7.172	19.044
638	7.141	19.041
639	7.119	19.038

640	7.093	19.021
641	7.067	19.018
642	7.042	19.024
643	7.006	19.014
644	6.982	19.018
645	6.949	19.019
646	6.922	19.007
647	6.902	19.002
648	6.874	19
649	6.856	18.999
650	6.833	19.001
651	6.808	19.001
652	6.792	18.997
653	6.763	18.994
654	6.741	18.985
655	6.724	18.981
656	6.692	18.983
657	6.675	18.977
658	6.647	18.969
659	6.623	18.96
660	6.604	18.954
661	6.577	18.938
662	6.561	18.928
663	6.541	18.926
664	6.517	18.93
665	6.504	18.928
666	6.477	18.929
667	6.46	18.933
668	6.44	18.934
669	6.427	18.932
670	6.411	18.921
671	6.402	18.931
672	6.392	18.941
673	6.374	18.938
674	6.354	18.932
675	6.339	18.934
676	6.317	18.928
677	6.304	18.924
678	6.293	18.928
679	6.272	18.929
680	6.259	18.946
681	6.248	18.96
682	6.232	18.954

683	6.226	18.948
684	6.215	18.951
685	6.204	18.943
686	6.194	18.933
687	6.187	18.942
688	6.172	18.947
689	6.162	18.939
690	6.15	18.941
691	6.134	18.943
692	6.125	18.938
693	6.118	18.939
694	6.117	18.954
695	6.108	18.959
696	6.101	18.974
697	6.088	18.988
698	6.078	18.985
699	6.064	18.98
700	6.047	18.97
701	6.039	18.954
702	6.032	18.945
703	6.018	18.948
704	6.013	18.953
705	6.008	18.958
706	6.008	18.955
707	6.002	18.96
708	5.998	18.975
709	5.995	18.976
710	5.986	18.975
711	5.985	18.972
712	5.989	18.976
713	5.976	18.985
714	5.977	19.003
715	5.973	19.016
716	5.962	19.036
717	5.967	19.048
718	5.965	19.053
719	5.96	19.061
720	5.961	19.081
721	5.961	19.092
722	5.96	19.099
723	5.96	19.115
724	5.958	19.125
725	5.958	19.133

726	5.958	19.138
727	5.958	19.145
728	5.96	19.15
729	5.96	19.15
730	5.96	19.153
731	5.96	19.16
732	5.96	19.164
733	5.959	19.169
734	5.96	19.164
735	5.961	19.169
736	5.959	19.171
737	5.959	19.176
738	5.957	19.176
739	5.957	19.176
740	5.958	19.181
741	5.957	19.181
742	5.956	19.183
743	5.955	19.185
744	5.955	19.182
745	5.956	19.188
746	5.956	19.191
747	5.957	19.196
748	5.958	19.196
749	5.958	19.194
750	5.96	19.198
751	5.96	19.199
752	5.961	19.203
753	5.964	19.203
754	5.965	19.201
755	5.966	19.202
756	5.967	19.201
757	5.971	19.204
758	5.975	19.203
759	5.977	19.203
760	5.98	19.208
761	5.983	19.207
762	5.987	19.212
763	5.99	19.214
764	5.993	19.219
765	5.998	19.218
766	6.004	19.221
767	6.008	19.221
768	6.014	19.222

769	6.018	19.227
770	6.022	19.224
771	6.027	19.224
772	6.031	19.229
773	6.036	19.233
774	6.042	19.235
775	6.048	19.234
776	6.053	19.237
777	6.058	19.239
778	6.065	19.242
779	6.069	19.244
780	6.076	19.241
781	6.084	19.245
782	6.091	19.246
783	6.098	19.243
784	6.105	19.244
785	6.112	19.241
786	6.119	19.239
787	6.126	19.239
788	6.134	19.24
789	6.14	19.242
790	6.149	19.239
791	6.158	19.236
792	6.167	19.235
793	6.179	19.234
794	6.188	19.238
795	6.196	19.237
796	6.208	19.235
797	6.217	19.231
798	6.227	19.228
799	6.238	19.228
800	6.247	19.228
801	6.257	19.227
802	6.267	19.225
803	6.275	19.226
804	6.287	19.225
805	6.297	19.223
806	6.308	19.22
807	6.318	19.219
808	6.329	19.222
809	6.339	19.222
810	6.35	19.22
811	6.363	19.211

812	6.374	19.206
813	6.383	19.203
814	6.394	19.201
815	6.403	19.2
816	6.415	19.196
817	6.425	19.185
818	6.433	19.184
819	6.444	19.18
820	6.452	19.178
821	6.462	19.178
822	6.471	19.176
823	6.483	19.169
824	6.488	19.167
825	6.496	19.161
826	6.499	19.142
827	6.51	19.122
828	6.515	19.121
829	6.526	19.117
830	6.537	19.1
831	6.55	19.091
832	6.549	19.102
833	6.556	19.085
834	6.551	19.084
835	6.565	19.099
836	6.578	19.084
837	6.596	19.079
838	6.598	19.077
839	6.593	19.083
840	6.614	19.084
841	6.617	19.09
842	6.63	19.086
843	6.63	19.032
844	6.641	19.022
845	6.658	19.021
846	6.668	18.994
847	6.664	19.003
848	6.68	19.023
849	6.704	19.022
850	6.724	19.014
851	6.745	19.014
852	6.768	18.994
853	6.776	18.992
854	6.799	19.018

855	6.819	19.043
856	6.846	19.049
857	6.874	19.036
858	6.89	19.031
859	6.912	19.013
860	6.915	19.01
861	6.921	19.003
862	6.939	18.993
863	6.955	19.02
864	6.971	19.011
865	6.987	19.009
866	6.988	18.992
867	6.99	18.982
868	6.993	18.996
869	7.011	18.997
870	7.025	18.995
871	7.034	18.987
872	7.05	18.988
873	7.058	18.981
874	7.072	18.976
875	7.094	18.976
876	7.12	18.978
877	7.14	18.991
878	7.155	18.995
879	7.173	19.003
880	7.196	19.015
881	7.217	19.028
882	7.232	19.053
883	7.248	19.066
884	7.265	19.082
885	7.286	19.087
886	7.302	19.106
887	7.32	19.113
888	7.348	19.117
889	7.368	19.12
890	7.385	19.12
891	7.406	19.117
892	7.424	19.12
893	7.445	19.117
894	7.462	19.116
895	7.483	19.12
896	7.505	19.111
897	7.519	19.107

898	7.534	19.113
899	7.553	19.112
900	7.567	19.111
901	7.588	19.11
902	7.601	19.108
903	7.616	19.108
904	7.635	19.117
905	7.647	19.116
906	7.661	19.114
907	7.669	19.113
908	7.678	19.117
909	7.697	19.112
910	7.703	19.115
911	7.715	19.114
912	7.725	19.116
913	7.734	19.112
914	7.75	19.099
915	7.762	19.088
916	7.783	19.076
917	7.802	19.06
918	7.819	19.05
919	7.831	19.029
920	7.843	19.016
921	7.856	18.992
922	7.866	18.97
923	7.884	18.944
924	7.892	18.925
925	7.903	18.914
926	7.916	18.9
927	7.927	18.877
928	7.938	18.852
929	7.948	18.837
930	7.962	18.823
931	7.981	18.797
932	8	18.78
933	8.014	18.77
934	8.028	18.753
935	8.045	18.741
936	8.063	18.722
937	8.08	18.701
938	8.103	18.688
939	8.126	18.68
940	8.151	18.668

941	8.172	18.659
942	8.199	18.654
943	8.228	18.65
944	8.262	18.647
945	8.3	18.65
946	8.337	18.654
947	8.375	18.661
948	8.412	18.673
949	8.452	18.69
950	8.495	18.71
951	8.538	18.733
952	8.59	18.758
953	8.635	18.79
954	8.686	18.825
955	8.734	18.858
956	8.79	18.895
957	8.843	18.939
958	8.902	18.984
959	8.958	19.029
960	9.016	19.077
961	9.075	19.121
962	9.132	19.171
963	9.189	19.219
964	9.248	19.265
965	9.305	19.314
966	9.358	19.362
967	9.408	19.41
968	9.46	19.45
969	9.516	19.493
970	9.567	19.529
971	9.615	19.563
972	9.657	19.6
973	9.698	19.63
974	9.734	19.656
975	9.766	19.673
976	9.792	19.681
977	9.814	19.688
978	9.828	19.687
979	9.837	19.677
980	9.834	19.663
981	9.827	19.641
982	9.812	19.614
983	9.789	19.573

984	9.759	19.525
985	9.721	19.465
986	9.675	19.403
987	9.627	19.334
988	9.569	19.254
989	9.504	19.165
990	9.437	19.074
991	9.359	18.973
992	9.279	18.865
993	9.197	18.753
994	9.114	18.634
995	9.031	18.51
996	8.949	18.388
997	8.869	18.261
998	8.79	18.132
999	8.716	18.004
1000	8.652	17.878
1001	8.597	17.754
1002	8.554	17.63
1003	8.526	17.516
1004	8.508	17.404
1005	8.508	17.307
1006	8.523	17.218
1007	8.56	17.138
1008	8.616	17.071
1009	8.698	17.015
1010	8.804	16.972
1011	8.932	16.942
1012	9.087	16.926
1013	9.268	16.932
1014	9.475	16.949
1015	9.708	16.984
1016	9.97	17.033
1017	10.257	17.102
1018	10.561	17.189
1019	10.888	17.296
1020	11.23	17.419
1021	11.593	17.563
1022	11.968	17.721
1023	12.357	17.901
1024	12.755	18.094
1025	13.161	18.305
1026	13.573	18.532

1027	13.988	18.776
1028	14.404	19.031
1029	14.82	19.3
1030	15.23	19.58
1031	15.635	19.874
1032	16.024	20.169
1033	16.398	20.476
1034	16.755	20.786
1035	17.09	21.104
1036	17.399	21.421
1037	17.677	21.741
1038	17.922	22.057
1039	18.135	22.372
1040	18.312	22.676
1041	18.449	22.971
1042	18.548	23.253
1043	18.607	23.532
1044	18.626	23.795
1045	18.603	24.043
1046	18.537	24.271
1047	18.432	24.485
1048	18.286	24.678
1049	18.105	24.846
1050	17.883	24.992

Fig. 4

wavelength (nm)	Lateral collection length (nm)	
	CdS/CIGS/Mo	CIGS/Mo
600	3699	2180
700	3739	2225
800	3744	2233
900	3429	2131
970	3517	1654
1000	2930	1410
1025	2125	1075
1050	972	462
